# Ethics preparedness: facilitating ethics review during outbreaks - recommendations from an expert panel

**DOI:** 10.1186/s12910-019-0366-x

**Published:** 2019-05-06

**Authors:** Abha Saxena, Peter Horby, John Amuasi, Nic Aagaard, Johannes Köhler, Ehsan Shamsi Gooshki, Emmanuelle Denis, Andreas A. Reis, the ALERRT-WHO Workshop, Flore Gangbo, Germaine Minoungou Compaore, Olive Nicole Ngaba, Narcisse P. Komas, Felicien Munday, Leslie Mawuli Aglanu, Kofi Wellington, Oumou Maiga-Ascofaré, Oumou Younoussa Bah-Sow, Alpha Ahmadou Diallo, Ousmane Souaré, Louis Penali, Gloria T. Mason, Joseph Mfutso-Bengo, Abdou Doumbia, Zubairu Iliyasu, Morenike Oluwatoyin Folayan, El Hadji Mbaye, Birahim Pierre Ndiaye, Aissatou Touré, Pape Touré, Ousman Nyan, Hend Bouacha, Irene Semakula Seryazi, Mahmood uz Jahan, Nijmeh Mohammed Hussein Al-Atiyyat, Aarati Shah, Ahmad Aasim, Leonardo De Castro, Gabriela Marodin, Marta Ascurra, Eleni Rethymiotaki, Eimantas Peicius, Eileen C. Farnon, Fabien Nicolas Taieb, Bernard Taverne, Catrin E. Moore, João Rangel de Almeida, Hugh Whittall, Carla Saenz, Vasee Sathiyamoorthy, Raffaella Ravinetto

**Affiliations:** 1 Global Health Ethics Team, World Health Organization, Geneva, Switzerland; 2 University of Geneva, Geneva, Switzerland; 3 Centre for Tropical Medicine and Global Health, University of Oxford, Oxford, UK; 4 Kumasi Center for Collaborative Research in Tropical Medicine, Kwame Nkrumah University of Science and Technology, Kumasi, Ghana; 5 Ethics Committees, Ministry of Health, Wellington, New Zealand; 6 Medical Ethics and History of Medicine Research Center, Tehran University of Medical Sciences, Tehran, Iran; 7 Institutional Review Board, Institute of Tropical Medicine, Antwerp, Belgium

**Keywords:** Research ethics, Ethics review, Rapid review, Pre-review, Infectious disease outbreaks, Low- and middle-income countries

## Abstract

**Background:**

Ensuring that countries have adequate research capacities is essential for an effective and efficient response to infectious disease outbreaks. The need for ethical principles and values embodied in international research ethics guidelines to be upheld during public health emergencies is widely recognized. Public health officials, researchers and other concerned stakeholders also have to carefully balance time and resources allocated to immediate treatment and control activities, with an approach that integrates research as part of the outbreak response. Under such circumstances, research “ethics preparedness” constitutes an important foundation for an effective response to infectious disease outbreaks and other health emergencies.

**Main text:**

A two-day workshop was convened in March 2018 by the World Health Organisation Global Health Ethics Team and the African coaLition for Epidemic Research, Response and Training, with representatives of National Ethics Committees, to identify practical processes and procedures related to ethics review preparedness. The workshop considered five areas where work might be undertaken to facilitate rapid and sound ethics review: preparing national ethics committees for outbreak response; pre-review of protocols; multi-country review; coordination between national ethics committees and other key stakeholders; data and benefit sharing; and export of samples to third countries.

In this paper, we present the recommendations that resulted from the workshop. In particular, the participants recommended that Ethics Committees would develop a formal national standard operating procedure for emergency response ethical review; that there is a need to clarify the terminology and expectations of pre-review of generic protocols and agree upon specific terminology; that there is a need to explore mechanisms for multi-country emergency ethical consultation, and to establish procedures for communication between national ethics committees and other oversight bodies and public health authorities. In addition, it was suggested that ethics committees should request from researchers, at a minimum, a preliminary data sharing and sample sharing plan that outlines the benefit to the population from which data and samples are to be drawn. This should be followed in due time by a full plan.

**Conclusion:**

It is hoped that the national ethics committees, supported by the WHO, relevant collaborative research consortia and external funding agencies, will work towards bringing these recommendations into practice, for supporting the conduct of effective research during outbreaks.

## The WHO-ALERRT workshop

To respond to infectious disease outbreaks and other health emergencies, not only do countries need adequate capacities for surveillance and infection control, but also adequate research capacities [1–3]. Knowledge generated through high-quality research in anticipation of, in the midst of, and after an outbreak is critical to prevent illness, disability and death and to support recovery, including that of health systems [4]. Rapid sharing of new information arising from research has the potential to influence public health decision-making, provided that the responsible stakeholders have the will, skills and competencies to integrate such information into decision making. While the need to conduct pertinent, scientifically and ethically sound research during outbreaks has long been recognized, its implementation remains challenging, particularly when it comes to the enforcement of sound research ethics standards [4–24]. “Ethics preparedness” has been identified as an important foundation for an effective public health response to infectious disease outbreaks and other health emergencies, including for research [25]. The ethics platform developed by WHO can assist stakeholders in integrating ethical considerations into many aspects of the outbreak response [26]. Several ethics workshops have been conducted in the recent past by WHO and others, for affected and at-risk low- and middle-income countries (LMICs) to raise awareness and understanding of the normative ethical concerns. But countries that have hosted and implemented research during outbreaks (such as Lassa fever in Nigeria, and the ongoing Ebola virus disease epidemic in the Democratic Republic of Congo) have highlighted the need for greater support and guidance to national research ethics committees, to develop practical and effective actions for achieving a robust but rapid research ethics review. To address such challenges, a two-day workshop was convened in March 2018 by the WHO Global Health Ethics Team in partnership with the African coaLition for Epidemic Research, Response and Training (ALERRT), a multi-disciplinary research consortium which responds to epidemics across sub-Saharan Africa.

The workshop aimed to identify practical processes and procedures to facilitate rapid but robust ethics review of research proposed during an infectious disease outbreak; and to formulate recommendations that may assist the timely implementation of research, to support national and international outbreak preparedness and response. Fifty-one representatives of national ethics committees (NECs) and national research ethics committees (NRECs) from 29 countries participated, some of which have experience reviewing research projects during epidemics and other health emergencies. Participants were from Africa (*n* = 25), Asia (*n* = 7), Europe (*n* = 15), the Americas (*n* = 3) and Oceania (*n* = 1), including representatives of the World Health Organization (WHO) Global Health Ethics Team, Pan American Health Organization (PAHO) regional program on bioethics, and ALERRT.

Noteworthy, substantive ethical issues were already being investigated by the Nuffield Council of Bioethics by means of a broader and inclusive call for evidence on “Research in global health emergencies: ethical issues” (http://nuffieldbioethics.org/project/global-health-emergencies). Therefore, building on other groups’ previous and ongoing work on substantive ethics issues, in this workshop, WHO and ALERRT placed the focus on ethics review *processes and procedures*. While the review of social science research during epidemics is an important area of enquiry, it was decided to focus on a limited number of issues relevant to health research, to allow for a pertinent and productive discussion within a restricted time frame. Based on past experience and review of literature, the workshop considered five priority areas requiring additional attention, and where work might be undertaken to facilitate rapid and sound ethics review during outbreaks: 1 - Preparing N(R)ECs for outbreak response; 2 - Pre-review of (generic) protocols; 3 - Streamlining the review of multi-country protocols; 4 - Interaction/Coordination between N(R)ECs and other research oversight bodies & public health authorities; 5 - Data, sample and benefit sharing.

The workshop included plenary presentations, panel discussions and small group discussions around specific issues and challenges, with the aim of generating agreement among the participants on the challenges posed by the five identified themes, and practical actions to address those challenges. A facilitator and a rapporteur were identified for each group discussion, and a rapporteur was appointed for each day of the workshop. The facilitators and rapporteurs of the group discussions helped to capture key deliberations and outcomes that were presented and further discussed at plenary sessions. The plenary sessions were specifically convened at the end of each session to enable participants to discuss and agree on one or more draft recommendations. The draft recommendations were further discussed in a plenary session towards the end of the Workshop. Draft proceedings were written and circulated twice by email to all the attendants, first for input and afterwards for informal approval or non-objection within the plenum. The final recommendations from each session, were made available on the ALERRT website in English, French and Arabic as of 12 June 2018 [27], and are summarized in Table 1. For each recommendation, a corresponding “suggested action” has been formulated.

**Table 1:** Recommendations and suggested actions from the Workshop

**•** ***Recommendation 1*** - A national standard operating procedure (SOP)for emergency response ethical review should be developed by and adopted by (N)RECs and/or in-country competent authority. *Suggested action* - Regional workshops should be held with the aim of drafting a “model SOP” for emergency response ethical review for adaptation and adoption at country level and/or through sub-regional mechanisms.	
**•** ***Recommendation 2*** - The group recommended clarifying the terminology and expectations of pre-review, pre-approval, generic protocols etc., and proposing specific terminology that could be agreed upon (in multiple languages when appropriate, and at a minimum in English, French and Spanish). *Suggested action* - A paper should be drafted that explores and clarifies the terminology and expectations of pre-approval, and which proposes specific terminology.	
**•** ***Recommendation (3a)*** - Mechanisms should be explored for (regional) multi-country emergency ethical consultation to support rapid review at country level. *Suggested action* - WHO should lead a process for a consultation on “multi-country rapid ethics review” in competent and legitimate fora, involving sub-regional mechanisms as appropriate.	
**•** ***Recommendation (3b)*** - The role of WHO in multi-country ethical review in Public Health Emergencies of International Concern (PHEICs) and “other similar emergencies” (as for wording used during the workshop) should be clarified *Suggested action* - WHO should define the scope of “other similar emergencies” and carry out regional consultations on the proposal for WHO supported review of multi-country research during PHEIC and “other similar emergencies”.	
**•** ***Recommendation 4*** - Emergency SOPs should include procedures for communications between (N)RECs and other key national stakeholders e.g. National Regulatory Authorities, Public health authorities, and between (N)RECs and relevant research stakeholders. *Suggested action* - Procedures for communications between (N)RECs and other key stakeholders should be specified in the “model SOP” (see Action 1).	
**•** ***Recommendation 5a*** - (N)RECs should request at a minimum, as part of a broader benefit sharing plan, a *preliminary* data sharing and sample sharing plan that outlines plans for how the results of the research and the samples will be made available for public health and other purposes, and first and foremost for the benefit of the population (countries) from which they were drawn. It is acceptable for the preliminary data and sample sharing plan to contain uncertainties when the health emergency is rapidly evolving and the need for research is urgent. *Suggested action* - The requirement for submission of preliminary and full data and sample sharing plans should be specified in the “model SOP” (see Action 1).	
**•** ***Recommendation 5b*** - (N)RECs should require applicants to submit a full data sharing and sample sharing plan within a defined period that lays out in detail how the results of the research and the samples will be made available for public health and other purposes, and first and foremost for the benefit of the population (countries) from which they were drawn. *Suggested action* - The requirement for submission of preliminary and full data and sample sharing plans should be specified in the “model SOP” (see Action 1).	
**•** ***Recommendation 6*** *-* (N)RECs should engage with relevant national authorities (MoH, environment e.g. Nagoya protocol, Legal experts etc.) to ensure that MTAs are ethically sound. *Suggested action* - An inventory should be conducted of resources used by (N)RECs related to MTAs.	

## The five sessions of the WHO-ALERRT workshop

The discussion during the entire workshop was guided by the commonly accepted notion that during public health emergencies, the ethical principles and values embodied in international research ethics guidelines must be upheld [7]. Recognizing the complexities and uncertainties during an outbreak, participants agreed that the exacerbated vulnerabilities of populations in such a situation automatically puts them at a greater risk of harm from research conducted during this time. N(R)ECs need to be cognizant of this during their deliberations and discussions.

### Session 1 - preparing RECs for outbreak response

In 2016, a WHO guidance document on ethical issues in outbreaks explicitly recommended that mechanisms must be developed to ensure rapid ethics review without undermining any of the substantive protections that a robust ethics review is designed to provide [7]. However, the guideline does not elaborate on what those mechanisms could be; and it is not clear for how long ethics review bodies may sustain the accelerated review timelines under emergency conditions [8, 28–30]. To the best of our knowledge, explicit mechanisms for rapid ethics review have not been put in place yet at the national level, or even piloted in any country. In order to highlight the challenges to a rapid ethics review, in the first session representatives of the WHO Ethics Review Committee (WHO ERC), the Liberian National Ethics Committee and the Institutional Review Board (IRB) of the Institute of Tropical Medicine (ITM) in Antwerp, (Belgium) shared their experiences of reviewing research protocols implemented during the 2014–2016 EVD outbreak in West Africa. The WHO ERC found it useful to follow SOPs specifically developed for accelerated review of proposals received during an emergency; these included the formation of a sub-committee, dedicated to reviewing protocols on tight timelines. Prior training of ERC members on specific issues relevant for epidemics, and the availability of experts as advisors, was found to be helpful by the Liberian Ethics Committee. The ITM IRB and researchers observed that there is much more to be done to harmonize and streamline the multiple ethical reviews of the same research protocol [30]. Subsequently, participants worked in small groups around case-vignettes (i.e., brief scenarios developed for the purpose of the workshop), in order to discuss the barriers to implementation of the existing normative guidelines, and what would help the N(R)ECs to be better prepared for providing a rapid and robust review of research during an epidemic.

There was agreement among participants that rapid review of research proposals and protocols should not risk reducing the quality of ethics committee decision-making, nor lead to rushed or superficial decision-making, nor lead to the approval of poor-quality or non-pertinent research.

Participants also agreed that following on the example provided by the WHO ERC, it would be helpful that a Standard Operating Procedures (SOP) for “emergency response ethical review” was developed and adopted by each N(R)EC; such SOP should include provisions to establish a multi-disciplinary standing sub-committee composed of N(R)EC members who would volunteer upfront to be called upon in times of emergency to conduct rapid reviews, and would have sufficient review training and expertise as to ensure that the quality of the review is not compromised despite the time pressure induced by the outbreak.

Having a SOP would promote consistency and efficiency of the review, since the procedures would be pre-established, and could be rapidly implemented. In addition, it would promote accountability, by making processes clear and transparent. While it is recognized that there may be legitimate reasons for withholding certain types of information in any public health emergency (for instance, if it jeopardizes national security, unnecessarily violates confidentiality and privacy, causes stigmatization, or might lead to behaviours resulting in increased spread of the disease) [31], transparency remains of paramount importance in the context of ethics review, as the N(R)ECs may prioritize research about the outbreak, which could negatively impact other key research projects on topics unrelated to the outbreak. Rapid sharing of information could also raise ethical risks in relation to use of data. Therefore, effective management of public health emergencies demands open and transparent public communication by N(R)ECs, and the upfront development of SOPs would be one such step. The group also recommended that this SOP would define the pre-set criteria that must be met for the sub-committee to be activated; and that it would include criteria to allow for rapid screening of all the received emergency protocols, based on the level of medical, social, psychological and any other risks associated with each proposed research. Research categorized as “high risk” would require more procedural protections, while research categorized as “lower risk” would require less procedural protections. It would also be helpful if the SOP would contain provisions to enable the N(R)EC to co-ordinate ethical oversight of all research conducted in the country during the emergency, rather than scattering the responsibility across a diversity of institutional ECs or IRBs. Participants suggested that N(R)ECs could consider pre-establishing the communication pathways to coordinate messaging from/to the different ECs and IRBs. This could be through the use of up-to-date email lists or a contact database. Further, N(R)ECs could consider supporting researchers during times of emergency to ensure that trust is built, good communication with communities is ensured and key-messages are shared directly with the population.

In the concluding session of the workshop, the participants developed the text of a recommendation that was completed with input from Session 5 and later finalised and approved by email (Recommendation 1 Table 1, and https://www.alerrt.global/content/ethics-preparednesss-facilitating-ethics-review-during-outbreaks).

### Session 2 - pre-review of (generic) protocols

The pre-review of “generic” protocols by ethics committees prior to an outbreak has been proposed as a mechanism to facilitate timely implementation of research when an outbreak does occur. Whether such an approach actually facilitates the implementation of research is yet to be established [32]. WHO’s and PAHO’s ERCs provide pre-review of protocols developed for use during an epidemic, while the Ethics Review Board (ERB) of Médecins Sans Frontières (MSF) encourages submission of “generic” protocols, to be further rapidly adapted to a specific context when an outbreak starts [33, 34]. In all cases, once the occurrence of an outbreak is established, the final contextualized protocol can be submitted for expedited review. However, to-date there is a lack of published examples of the process of pre-review of emergency protocols.

Based on the above, this session was organized as a plenary discussion, based on experience shared by an international research group and of one ethics review board with a specific mandate (that of the medico-humanitarian organization MSF). A moderator gathered opinions of participants on challenges faced by N(R)ECs when confronted with time constraints to review protocols to be implemented during an outbreak; and opinions on the advance review of “generic” protocols for conducting research in outbreak conditions, as a possible solution. Initially, the expression “pre-approval” of protocols was used instead of “pre-review”. However, participants expressed concern that “pre-approval” being a very specific, official term, suggested challenging ethics committees to provide a definitive decision. “Pre-approval” might also suggest a green light to proceed with the research, which would be inappropriate, since a “generic” protocol still lacks important contextual details, such as on the study location, study population, local standard of care and context-related risks. Therefore, the terminology was reconsidered to describe the intent of the suggested procedure, that is a prior review of a generic protocol, to provide input ahead of an outbreak on the research background, rationale, objectives, methodology, outcomes, informed consent procedures etc. However, full ethics “approval” can only be granted once the context is known, and the generic protocol has been contextualized for the specific location(s) and community(ies). Therefore, the wording “pre-review” was considered to be more appropriate. Other expressions that were suggested but eventually discarded included “pre-consider” or “conditional approval”.

Participants further discussed the timing for the “final approval” of the protocol. They agreed that the process being discussed was the prior review in “peacetime” of a full or partial generic protocol so that the (N)REC may provide advice ahead of an outbreak. This is to be followed by the expedited review of a final, contextualized protocol that incorporates detailed information and considerations on the location(s), and context-related aspects such as communities’ vulnerability, community engagement, standard of care.

Provided that the full “ethical approval” to proceed to research implementation may only be granted to a contextualized final protocol, participants were of the opinion that a “pre-review” could help to accelerate the review of the final protocol, and make the process of ethics review more efficient and effective, as most key ethical issues would have already been considered during the pre-review process, and the (N)REC would be already familiar with the protocol. Acknowledging the possibility that the same wording may be interpreted differently by different stakeholders, the group recommended further developing and proposing specific terminology that could be agreed upon to better focus the conversation and expectations of “pre-review” of protocols.

(Recommendation 2, Table 1; and https://www.alerrt.global/content/ethics-preparednesss-facilitating-ethics-review-during-outbreaks)

### Session 3 - streamlining the review of multi-country protocols

Research during an epidemic often involves many organizations and countries, and multiple reviews. These can strengthen trial protocols but, in the absence of coordination and communication among ECs and IRBs, they can also contribute to avoidable delays in trial implementation. To contribute to understanding how such coordination and collaboration could work in practice, the participants worked in small groups using a case-vignette to identify the challenges related to the review of multi-country protocols, and to propose mitigating measures.

The group noted that multi-country protocols should not be conflated with multi-site protocols within the same country. Engagement and communication between each N(R)EC and the local ECs and IRBs with the aim to provide a single review in the country was considered as critical, and these aspects could be addressed in the national SOPs discussed in Section 1.

Participants generally agreed that multiple reviews of multi-country protocols present an opportunity for complementariness through sharing of perspectives of different countries, with the potential for mutual learning. However, they also recognized that if not accompanied by clear communication and exchange across the different review bodies, multiple reviews may introduce contradictions and redundancies, and present a challenge to rapid review processes. Needless to say, the consequences of contradictions, redundancies and delays would be particularly harmful for research conducted during outbreaks and other public health emergencies. There was also agreement among participants that to enhance the positive aspects of multiple reviews and decrease associated risks, harmonization of the review process could be considered. Such harmonization may take place at different levels of formality. For example, facilitating direct informal discussion between the reviewing bodies could reduce contradictions, foster consistency across reviews, increase learning and dialogue between review bodies, and ultimately build shared good practices. Participants cautioned that informal discussions could risk compromising transparency if not documented, but on the other hand, recognized that documentation of informal discussions could have unintended consequences if they were taken out of context. It was therefore considered important to establish procedures to document informal communications as well.

Moving toward more formal mechanisms to foster harmonization, the participants suggested that regional mechanisms for multi-country emergency ethical consultation to support rapid N(R)EC review could be explored. This option would be strongly recommended where an appropriate and legitimate “umbrella” entity exists. The African Vaccine Regulatory Forum (AVAREF) was given as an example of a successful platform for multi-country review. At the same time, it was noted that an initial consultation process that brings together representatives of the different N(R)ECs cannot replace the national review. Its aim would be to achieve a process of harmonisation of criteria and procedures across the different N(R)ECs, to facilitate the submission and allow some acceleration of multi-country review, while the final review and definitive national approval by each N(R)EC would preserve their independence. WHO, as a neutral and trusted partner with the ability to convene various stakeholders round the table, could play a critical role in facilitating a multi-country ethics review platform.

During the debate, there was some uncertainty as to whether or not such a harmonization process, including the role attributed to WHO and its regional offices, would be limited to research related to infectious diseases outbreaks, or whether it would also include other similar emergencies, and in particular Public Health Emergencies of International Concern (PHEICs).

(Recommendations 3a and 3b, Table 1; and https://www.alerrt.global/content/ethics-preparednesss-facilitating-ethics-review-during-outbreaks).

### Session 4 - interaction/coordination between N(R)ECs and other research oversight bodies & public health authorities

The need to have effective communication between research ethics committees and public health and disaster relief agencies during research carried out during a humanitarian crisis and post disaster has been identified in earlier literature [24, 30, 34], but mechanisms to do so have not been elaborated. During this session, the representatives of several ethics committees from countries where there had been recent past epidemics, including of Ebola and Zika viruses, shared their experience of what worked and what did not. In the plenary discussion, participants noted that research conducted during outbreaks and other similar emergencies requires rapid decision-making and action, as well as aligning research activities with public health decision-making.

It was suggested that, in order to facilitate rapid and sound decisions around research activities, and ensure systematic and consistent alignment between research and public health priorities and activities, N(R)ECs may consider having written procedures that guide direct communication with other key stakeholders. These proposed procedures do not only include the concerned national bodies such as public health authorities and national regulatory authorities (NRAs), but also actors involved in specific research projects, such as the research advisory bodies, and Data and Safety Monitoring Boards (DSMBs) appointed by the sponsors of high-risk clinical trials for independent supervision. This inclusive approach could help optimize coordination across all these key-actors, foster trust, as well as facilitate a rapid and transparent communication of changing risk benefits as the epidemic evolves. The group recognized that in the interest of transparency, this would require a consultation with all stakeholders on the best way to implement the communication plan, as well as training of all concerned in effective and quality communication. Some participants suggested that the DSMBs may include a qualified member of the REC as an observer. (Recommendation 4, Table 1; and https://www.alerrt.global/content/ethics-preparednesss-facilitating-ethics-review-during-outbreaks).

### Session 5 - data, sample and benefit sharing

Rapid data sharing is critical during an unfolding health emergency [35, 36]. The transparent and timely dissemination of such data through peer-reviewed journals and accompanying (online) data sets is vital for decision-makers. Ethics committees clearly have a role in supporting this vital function, but this requires that the skills and knowledge needed for taking on this key-responsibility are built ahead of the epidemic.

To contribute to address such challenges, a plenary presentation based on the WHO guidance on research data and sample sharing was followed by the sharing of experience from LMICs in different geographic regions, and by a panel discussion. The participants agreed that rapid sharing of new information arising from research has the potential to influence public health decision making in any on-going, as well as in future outbreaks. Thus, a blanket decision not to share information would be unethical. However, sharing of research datasets and samples raised complex ethics questions, such as how will consent from individual patients be secured? How will the confidentiality of individuals and communities be protected? How will the scientific integrity of the research be protected from premature release of inconclusive results? Who will have the ownership or custodianship of datasets and samples? Who will benefit from the findings of any further research conducted on such data and samples [37, 38]? All participants recognized that these potential ethics challenges should be explicitly addressed upfront, but that at the early stages of an outbreak, the research sponsor and principal investigator(s) might not yet have certainty over what data and samples will be available and when. Consequently, they agreed on the need for N(R)ECs to adopt a pragmatic but sound approach to data and sample sharing.

In practice, when a research proposal is submitted (either as a generic proposal before an outbreak occurs, or as a contextualized final protocol in the early phases of the outbreak), N(R)ECs could consider a two-step approach. First, at a minimum, they would request a preliminary data and sample sharing plan that outlines how the results of the research, and the datasets and samples generated from the research, will be made available for public health and other purposes. Second, N(R)ECs would require applicants to re-submit for review and approval a full data sharing and sample sharing plan, within a defined period. The group suggested that when reviewing both the preliminary and the full data sharing plan sharing, N(R)ECs carefully distinguish between the sharing of the results of research, via a timely and adequate publication and communication plan, and the actual sharing of de-identified datasets and biological samples. The top-line interim results of research may be available before a de-identified, complete, cleaned and validated dataset is available, and conversely, a subset of informative data (e.g. on the baseline characteristics of research participants) may be available before the final conclusion of the research is reached.

However, there was uncertainty regarding the detailed process that could be followed to act on this recommendation, what capacities would be required and how the risks for the research participant/communities in relation to respecting their confidentiality and privacy would be allayed or mitigated. On a similar note, there was concern about maintaining respect for legitimate interests of countries and communities that share data and samples; about the adequate, transparent and fair governance mechanisms of databanks and biobanks; and about the forms that benefit sharing could and should take when questions on further research using shared data and samples arises. Considering the afore-mentioned, participants recommended that it could be helpful to develop upfront (i.e. before an outbreak occurs), standardized templates of Data Sharing Agreements and Material Transfer Agreements (MTAs). N(R)ECs would have a key role in ensuring that these templates incorporate ethical principles based on principles of fair partnerships, and that they are adequately implemented during and after the conclusion of the research. Importantly, despite the differences between collecting human data and samples and collecting natural genetic resources, some lessons could be drawn from the Nagoya protocol [39].

The issue of sharing of data and samples collected for public health purposes and not for research - though outside the scope of this workshop, was a major point of discussion. Participants agreed that N(R)ECs should play a facilitatory role in this regard and share existing resources with the public health agencies. First, healthcare facilities involved in the response to an outbreak could consider putting in place clear and transparent mechanisms for broad consent for use of de-identified data and samples collected for public health purposes for future research. Second, N(R)ECs could facilitate fair processes for development of national templates of MTAs to be used for samples collected outside a research environment. As a corollary to this, it was emphasized that MTAs are legal documents and need to be developed such that they have validity in the country of origin and the country of export, thus professionals with adequate expertise (going beyond the skills and capacities of N(R)ECs) will need to be involved in this process. Third, the participants recommended that the capacities to store and further use biological samples collected during outbreaks must be developed in the affected countries. To achieve this, the development of regional biobanks was highly recommended, in order to pool resources, and considering that legal requirements and cultural and contextual factors within a region are more likely to be consistent. This was an area where participants felt there was a need for more capacity strengthening for (N)RECs members, specifically in relation to intellectual property rights, benefit sharing and community engagement. (Recommendations 5a, 5b and 6, Table 1; and https://www.alerrt.global/content/ethics-preparednesss-facilitating-ethics-review-during-outbreaks).

## The recommendations of the WHO-ALERRT workshop

Six recommendations and related action emerged from the Workshop, the last two being related to Session 5 (see Table 1).

First, the participants recommended that to prepare for outbreak response, a *national standard operating procedure (SOP) for emergency response ethical review should be developed and adopted by N(R)ECs and/or in-country competent authority*. Such an ambitious goal could be initially achieved by means of regional workshops, while adaptation of the model SOP and adoption at country level could be dealt with through sub-regional mechanisms.

Second, while recognizing the importance of the pre-review of (generic) protocols for enhancing the quality and the timelines of the ethics review, they recommended clarifying the terminology and expectations of pre-review, pre-approval, generic protocols etc., and proposing specific terminology that could be agreed upon (in multiple languages when appropriate, and at a minimum in English, French and Spanish). The group suggested that a manuscript could be drafted that explores and clarifies the terminology and expectations of pre-approval, and which proposes specific terminology.

Third, the participants agreed that there is a need to explore mechanisms for (regional) multi-country emergency ethical consultation to support rapid review at country level. Corollary to this, they also underlined a need to clarify the role of WHO in multi-country ethical review in Public Health Emergencies of International Concern (PHEICs) and “other similar emergencies” (as for wording used during the workshop). To do so, it is hoped that the WHO may lead a process for a consultation on “multi-country rapid ethics review” in competent and legitimate fora, involving sub-regional mechanisms as appropriate; and that WHO may also define the scope of “other similar emergencies”, and carry out regional consultations on the proposal for WHO supported review of multi-country research during PHEIC and “other similar emergencies”.

Fourth, and linked to the first recommendation, it was recognized that during outbreaks there is a particularly urgent need to build interaction and coordination between N(R)ECs and other research oversight bodies & public health authorities. Consequently, it was recommended that the above “model SOP” would include explicit procedures for communications between N(R)ECs and other key national stakeholders e.g. national regulatory authorities, public health authorities, and between N(R)ECs and relevant research stakeholders. To do so, the procedures for communications between N(R)ECs and other key stakeholders should be specified in the “model SOP”.

Fifth, when it comes to ethical data and samples sharing, a two-step approach was recommended by the group. Initially, the N(R)ECs would request at a minimum, as part of a broader benefit sharing plan, a *preliminary* data sharing and sample sharing plan that outlines how the results of the research and the samples will be made available for public health and other purposes, and first and foremost for the benefit of the population (countries) from which they were drawn. It is acceptable for the preliminary data and sample sharing plan to contain uncertainties when the health emergency is rapidly evolving and the need for research is urgent. Further, the N(R)ECs would require applicants to submit a full data sharing and sample sharing plan, within a defined period, based on justice considerations and laying out in detail how the results of the research and the samples will be made available for public health and other purposes, and first and foremost for the benefit of the population (countries) from which they were drawn. The requirement for submission of preliminary and full data and sample sharing plans should also be specified in the “model SOP” (see Recommendation 1).

Sixth, when it comes to ethical management of bio-samples obtained during outbreak research, it was recommended that N(R)ECs engage with relevant national stakeholders (Ministries of Health, environmental bodies e.g. Nagoya protocol, legal experts etc.) to ensure that MTAs are ethically sound. An essential pre-requisite to do so, would be conducting an inventory of resources used by N(R)ECs related to MTAs.

The work that led to the development of these recommendations presents some limitations. As noted above, in view of the time constraints, the number of issues had to be limited, and we could not discuss other aspects of ethics review that could benefit from additional procedural guidance, such as adapted procedures for the ethics review of social science research and epidemiological surveys linked to public health response to outbreaks, building capacities of ethics committees in this regard, and the role of N(R)ECs in community engagement. In addition, we did not discuss if and how N(R)ECs may proactively monitor the implementation of their recommendations by researchers and sponsors. Such issues will benefit from additional discussion within this and other groups.

Despite this limitation, the recommendations emanating from this workshop imply that ethics review preparedness is an essential component of research preparedness for outbreaks and other similar emergencies. They emerged from the in-depth reflection of a group characterized by a great diversity of research-related roles and geographic representation, with first-hand experience from the Ebola outbreak in West Africa and from the Zika outbreak in Latin America. As such, they are highly relevant and hold potential to be globally applicable. In addition, they could also be useful in non-emergency research, such as for streamlining multi-country review, and for achieving fair sharing of data and bio-samples. Hopefully, the N(R)ECs supported by WHO, relevant collaborative research consortia and external funding agencies, will work towards bringing these recommendations into practice, for supporting the conduct of research efficiently and effectively during future infectious disease outbreaks.

## Abbreviations

ALERRT: African coaLition for Epidemic Research, Response and Training
DSMB: Data Safety Monitoring Board
EC: Ethics Committee
ERB: Ethics Review Board
ERC: Ethics Review Committee
EVD: Ebola Virus Disease
IRB: Institutional Review Board
ITM: Institute of Tropical Medicine
MSF: Médecins Sans Frontières
MTA: Material Transfer Agreement
NEC: National Ethics Committee
NRA: National Regulatory Authority
NREC: National Research Ethics Committee
PAHO: Pan American Health Organization
PHEICs: Public Health Emergencies of International Concern
SOP: Standard Operating Procedure
WHO: World Health Organization

## References

[1] Keusch GT, MacAdam KPWJ (2017). Clinical trials during epidemics. Lancet.

[2] Stellmach D, Beshar I, Bedford J (2018). Anthropology in public health emergencies: what is anthropology good for?. BMJ Glob Health.

[3] Peters DH, Tran NT, Adam T. Implementation Research in Health: A Practical Guide: World Health Organization; 2013. ISBN 978 92 4 150621 2. https://www.who.int/alliance-hpsr/alliancehpsr/_irpguide.pdf.

[4] Ni L, Manolio T, Patterson AP (2013). Research as a part of public health emergency response. N Engl J Med.

[5] Calain P, Fiore N, Poncin M, Hurst S (2009). Research ethics and international epidemic response: the case of Ebola and Marburg hemorrhagic fevers. Pub Health Ethics.

[6] World Health Organization (WHO). Ethical considerations for use of unregistered interventions for Ebola viral disease. Report of an advisory panel to WHO: WHO; 2014. Ethical issues related to study design for trials on therapeutics for Ebola virus disease WHO ethics working group meeting 20–21 October. Summary of Discussion. https://apps.who.int/iris/handle/10665/130997.

[7] World Health Organization (WHO). Guidance for managing ethical issues in infectious disease outbreaks: WHO; 2016. ISBN 978 92 4 154983 7. https://www.who.int/ethics/publications/infectious-disease-outbreaks/en.

[8] Schopper D, Ravinetto R, Schwartz L, et al. Research ethics governance in times of Ebola. Public Health Ethics. 2016. 10.1093/phe/phw039 First published online: November 1, 2016. 10.1093/phe/phw039 PMC5444563 28567113

[9] Nuffield Council of Bioethics. Conducting research and innovation in the context of global health emergencies: what are the ethical challenges? Notes of workshop held on 9 December 2016: 10:00–13:30 28 Bedford Square, London WC1B 3JS. http://nuffieldbioethics.org/wp-content/uploads/Global-health-emergencies-short-note.pdf.

[10] Upshur Ross, Fuller Jonathan (2016). Randomized controlled trials in the West African Ebola virus outbreak. Clinical Trials: Journal of the Society for Clinical Trials.

[11] Doussau A, Grady C (2016). Deciphering assumptions about stepped wedge designs: the case of Ebola vaccine research. Med Ethics.

[12] London AJ, Omotade OO, Mello MM, Keusch GT (2018). Ethics of randomized trials in a public health emergency. PLoS Negl Trop Dis.

[13] Carazo Perez S, Folkesson E, Anglaret X (2017). Challenges in preparing and implementing a clinical trial at field level in an Ebola emergency: A case study in Guinea, West Africa. PLoS Negl Trop Dis.

[14] Canario Guzmán JA, Espinal R, Baez J (2017). Ethical challenges for international collaborative research partnerships in the context of the Zika outbreak in the Dominican Republic: a qualitative case study. Health Res Policy Syst.

[15] O’Math’ una DP, ten Have HAMJ, Gordijn B (2014). Disasters. Handbook of global bioethics.

[16] Perakslis ED (2018). Using digital health to enable ethical health research in conflict and other humanitarian settings. Confl Heal.

[17] Merson L, Gaye O, Guerin PJ (2016). Avoiding data dumpsters — toward equitable and useful data sharing. N Engl J Med.

[18] Chen H, Pang T (2015). A call for global governance of biobanks. Bull World Health Organ.

[19] Richardson T, Johnston AM, Draper H (2017). A systematic review of Ebola treatment trials to assess the extent to which they adhere to ethical guidelines. PLoS One.

[20] Lanini S, Zumla A, Ioannidis JP (2015). Are adaptive randomised trials or non-randomised studies the best way to address the Ebola outbreak in West Africa?. Lancet Inf Dis.

[21] Busta ER, Mancher M, Cuff PA, McAdam K, Keusch G, National Academies of Sciences, Engineering, and Medicine, Health and Medicine Division, Board on Health Sciences Policy, Board on Global Health, Committee on Clinical Trials During the 2014–2015 Ebola Outbreak (2017). Integrating Clinical Research into Epidemic Response: The Ebola Experience.

[22] Ethics Working Group on ZIKA Research and Pregnancy (2017). Pregnant women and the Zika virus vaccine research agenda: ethics guidance on priorities, inclusion and evidence generation.

[23] Pan American Health Organization (2016). Zika ethics consultation: ethics guidance on key issues raised by the outbreak.

[24] Saenz C (2016). Zika virus: ethics preparedness for old and new challenges. Lancet GH.

[25] Presidential Commission for the Study of Bioethical Issues. ETHICS and EBOLA: Public Health Planning and Response (2015) Washington, D.C. Accessed on 25 Mar 2019 at https://bioethicsarchive.georgetown.edu/pcsbi/sites/default/files/Ethics-and-Ebola_PCSBI_508.pdf

[26] World Health Organization. Integrating ethics in infectious disease outbreaks. https://extranet.who.int/ethics/. Accessed 29 Apr 2019.

[27] “Ethics preparednesss”: Facilitating Ethics Review During Outbreaks. Recommendations arising from a joint ALERRT & WHO workshop. Dakar, Senegal, 20-21 March 2018. Accessed on 8 Oct 2018 at https://www.alerrt.global/content/ethics-preparednesss-facilitating-ethics-review-during-outbreaks.

[28] Alirol E, Kuesel AC, Guraiib MM (2017). Ethics review of studies during public health emergencies - the experience of the WHO ethics review committee during the Ebola virus disease epidemic. BMC Med Ethics.

[29] Aarons D (2018). Research in epidemic and emergency situations: a model for collaboration and expediting ethics review in two Caribbean countries. Dev World Bioeth.

[30] De Crop M, Delamou A, van Griensven J, Ravinetto R (2016). Multiple ethical review in north–south collaborative research: the experience of the Ebola-Tx trial in Guinea. Indian J Med Ethics.

[31] O’Malley P, Rainford J, Thompson A (2009). Transparency during public health emergencies: from rhetoric to reality. Bull World Health Organ.

[32] Rojek AM, Dunning J, Leliogdowicz A (2018). Regulatory and operational complexities of conducting a clinical treatment trial during an Ebola virus disease epidemic. Clin Infect Dis.

[33] Saxena Abha, personal communication. 2018.

[34] Schopper D, Dawson A, Upshur R (2015). Innovations in research ethics governance in humanitarian settings. BMC Med Ethics.

[35] Modjarrad K, Moorthy VS, Millett P (2016). Developing global norms for sharing data and results during public health emergencies. PLoS Med.

[36] Whitty CJM, Mundel T, Farrar J (2015). Providing incentives to share data early in health emergencies: the role of journal editors. Lancet..

[37] Hayden EC (2015). Biobank planned for Ebola samples. Nature.

[38] Tindana P, Molyneux S, Bull S, Parker M. 'It is an entrustment'. Broad consent for genomic research and biobanks in Sub-Saharan Africa. Dev World Bioeth. 2019;19(1):9-17. 10.1111/dewb.12178 PMC6624131 29063669

[39] Secretariat of the Convention on Biological Diversity. United Nations environmental Programme (2011). Nagoya protocol on access to genetic resources and the fair and equitable sharing of benefits arising from their utilization to the convention on biological diversity: text and annex.

